# ﻿Effects of habitat differences on the scatter-hoarding behaviour of rodents (Mammalia, Rodentia) in temperate forests

**DOI:** 10.3897/zookeys.1141.96883

**Published:** 2023-01-20

**Authors:** Dianwei Li, Jiahui Liu, Chengzhi Zhang, Yuwei Cao, Ming Gao, Zhimin Jin, Hongjia Shan, Hongwei Ni

**Affiliations:** 1 Heilongjiang Academy of Forestry, No. 134 Haping Road, Harbin, Heilongjiang 150081, China Heilongjiang Academy of Forestry Harbin China; 2 College of Life Sciences and Technology, Mudanjiang Normal University, No. 191 Wenhua Road, Mudanjiang, Heilongjiang 157011, China Mudanjiang Normal University Mudanjiang China; 3 College of Wildlife and Protected Area, Northeast Forestry University, No. 26 Hexing Road, Harbin 150040, China Northeast Forestry University Harbin China

**Keywords:** Habitat, rodents, scatter-hoarding, seed fate

## Abstract

To discover the differences in hoarding strategies of rodents for different seeds in different habitats, we labelled and released three different types of seeds, including *Pinuskoraiensis*, *Corylusmandshurica*, and *Quercusmongolica*, in temperate forests of northeastern China and investigated the fate of seeds in four different habitats that included a broad-leaved forest, mixed-forest edge, mixed forest, and artificial larch forest. Our research showed that the hoarding strategy of rodents was found to vary substantially in different habitats. The survival curves of seeds from different habitats showed the same trend, but the rates of consumption in different habitats varied. More than 50% of the seeds in the four habitats were consumed by the tenth day. It took 20 days to consume more than 70% of the seeds. The rate of consumption of *P.koraiensis* seeds reached 96.70%; 99.09% of the *C.mandshurica* seeds were consumed, and 93.07% of the *Q.mongolica* seeds were consumed. The seeds were consumed most quickly in the artificial larch forest. In general, most of the early seeds were quickly devoured. After day 20, the consumption gradually decreased. Rodents found the seeds in the artificial larch forest in a shorter average time than those in the other types of forests. The average earliest discovery time was 1.4 ± 0.9 d (1–3 d). The average earliest discovery time in all the other three habitats exceeded 7 d. The median removal times (MRT) was distributed around the seeds at 14.24 ± 10.53 d (1–60 d). There were significant differences in the MRT among different habitats. It was shortest in the artificial larch forest at 7.67 ± 6.80 d (1–28 d). In contrast, the MRT in the broad-leaved forest was the longest at 17.52 ± 12.91 d (4–60 d). There were significant differences in the MRT between the artificial larch forest and the other habitats. There was less predation of the three types of seeds at the mixed-forest edge, and the most seeds were dispersed. The rates of predation of the *P.koraiensis*, *C.mandshurica*, and *Q.mongolica* seeds were 28.33%, 15.83%, and 44.0%, and 59.17%, 84.17%, and 48.0% of the seeds were dispersed, respectively. The average dispersal distances of all the seeds were less than 6 m, and the longest distance recorded was 18.66 m. The dispersal distances and burial depths differed significantly among the four types of habitats. The distance of seed dispersal was primarily distributed in 1–6 m.

## ﻿Introduction

Food hoarding behaviour is a type of exceptional feeding activity of the rodents. It is regarded as a strategy to adapt to the periodic fluctuation of food resources, as well as the environment. (Vander Wall 1990, 2001; Lichti et al. 2015; Li et al. 2020). This benefits the rodent by rationally allocating limited food resources to manage food distribution and richness with the changes in time and space. Alternatively, triumphant hoarding is key for the survival and reproductive fitness of many types of species during periods of food scarcity (Vander Wall 2001; Lu and Zhang 2005; Lichti et al. 2015; Luna et al. 2016). In addition, in the forest ecosystem, many plants rely on animals as a manner of dispersing their seeds. The set of behaviours of hoarding animals, including the harvest, transport, and storage, affects the success of both seed germination and the survival of seedlings in a direct way (Vander Wall 2001; Lichti et al. 2015). The hoarding behaviour of rodents is one of the crucial processes that affects the dynamics, structure, spatial distribution, natural selection, and species diversity of plant populations and communities (Willson and Whelan 1990; Vander Wall 2001; Vander Wall and Beck 2011; Li et al. 2018).

A co-evolutionary reciprocity exists between many plants with large seeds and hoarding animals (Vander Wall 1990, 2001; Li and Zhang 2003). Some of the characteristics or habits of both parties contribute to the adaptation and strengthening of the mutualistic relationship during evolution. The hoarding behaviour is influenced by a variety of factors, which include the specialties of plant seeds, yield, distribution, temporal and spatial changes of food resources, and changes in environmental factors, such as climate and habitat structure (Preston and Jacobs 2005; Jenkins 2011; Lichti et al. 2015; Li et al. 2021). The temporal and spatial dependence of hoarding animals could be influenced by such factors as variation in the vegetation and the rhythm of activity of animals. Therefore, rodent behaviour can better reflect its adaptation to habitat selection and environmental changes.

The study site was located in the ecological region of the Zhangguangcai Mountains in north-eastern China. It is located at the northern end of Changbai Mountain. This zone is rich in forest vegetation resources, which is an important resource of species and seed bank. There are abundant types and quantities of rodents in forests. Rodents not only destroy forest resources by feeding on vegetation and seeds but also promote the regeneration of vegetation by dispersing and hoarding food (Vander Wall 2001, 2003; Lichti et al. 2015).

To further understand these issues to provide theoretical and practical guidance to explore the interaction between rodents and many large-seeded plants, this study labelled and released the seeds of *Pinuskoraiensis*, *Corylusmandshurica*, and *Quercusmongolica* in four different habitat types. They included a broad-leaved forest, mixed-forest edge, mixed forest, and artificial larch forest. The fate of seeds, predation, dispersal, and storage of the seeds by rodents were investigated, and the rules of utilization of the seeds by rodents and the habitat differences in natural environment were also investigated. The seeds were regularly investigated to understand the following: (1) the survival curves and consumption time of different habitat types; (2) the selection characteristics of different seeds in the same region; (3) the fate of released seeds; and (4) the characteristics of dispersal distance and burial depth of seeds. The results of this research should increase the theoretical basis to understand the influence of rodents on forest-tree seeds and provide a scientific basis for the renewal and protection of forest vegetation.

## ﻿Site and methods

### ﻿Study area and research site selection

The research was conducted in a forested area of the Sandao forest farm (44°40'N–44°45'N, 129°24'E–129°32'E, elevation 380–550 m a.s.l.), Mudanjiang City, from April to November 2019. The research area is located at the north end of Changbai Mountain in northeastern China at the east vein of the main ridge of Zhangguangcai Mountain. The climate is temperate and has a cold continental monsoon climate, four distinct seasons, and a hot rainy season. The highest temperature recorded here wass 37 °C. The lowest temperature recorded was −44.1 °C, and the annual average temperature is 2.3–3.7 °C. Four types of different habitats were selected for research in the field experiment. They included alternative broad-leaved forest plots with less human disturbance, mixed-forest edge, mixed forest, and artificial larch forest. Each plot was spaced more than 2 km from another plot. The composition of small rodents and the vegetation in the sample were investigated before the experiment was initiated. The rodents in forests were highly abundant and diverse. *Apodemuspeninsulae*, *A.agrarius*, and *Clethrionomysrufocanus* were the three most abundant seed predators/dispersers in the forest.

### ﻿Tagging and tracking of seeds

Healthy seeds of *P.koraiensis*, *C.mandshurica*, and *Q.mongolica* selected in the field study were marked using an electric drill whose bit is 0.5 mm in diameter, and holes were made at one end of the seed. A thin red plastic sheet was cut into a 3 cm × 1 cm rectangular piece, and a small hole was cut in the middle of the short side. The perforated seeds were connected to the plastic plate with a soft steel wire that was 0.3 mm in diameter and 8 cm long. The seed category, sample number, and seed number were marked on each label. This made the seeds easier to locate during research because the tags were exposed when the rodents ate the seeds or buried them in the ground, under dead branches or in shallow holes. The rodents could not bite off the steel wire. Therefore, this tagging method had no significant effect on their seed dispersal (Li et al. 2018, 2021).

### ﻿Release and investigation of the seeds

Food release stations in the forest were randomly spaced more than 50 m apart. A total of 20 seeds of each type were released from each planting for a total of 60 seeds. There were six release stations for each type of habitat and 120 seeds for each type, totalling 360 seeds. The studies were performed on days 1, 2, 3, 4, 6, 8, 12, 16, 20, 28, 36, 44, and 60 after release. The fate, characteristics and dispersal distance of the seeds were measured.

### ﻿Definition of seed fate

After finding the seeds, the animals chose different seeds based on their preferences and performed different operations, which led to different fates of the seeds. The fate of seeds released in field experiments was defined as previously described (Li et al. 2018, 2021):

1. Intact *in situ* (IS): seeds not eaten or removed from the station
2. Predation *in situ* (PS): seed kernels eaten at the seed station
3. Predation after removal (PR): seed kernels eaten after removal
4. Intact after removal (IR): seeds not eaten and abandoned on the surface of ground after removal
5. Hoarded after removal (HR): seeds buried in the soil or humus layer after removal
6. Missing after removal (MR): seeds removed but not found
7. Consumption: with the exception of intact *in situ* seeds, the fate of other seeds is defined as consumption by rodents.
8. Predation: predation *in situ* and predation after removal are defined as predation (Predation *in situ* + Predation after removal)
9. Dispersal: intact after removal, hoarded after removal, and missing after removal were defined as dispersal. However, there were no data records for the survey indicators of the missing seeds, so they could not be calculated during the inspection and comparison (Intact after removal + Hoarded after removal + Missing after removal)
10. Median removal time (MRT) of the seeds: the time at which 50% of the seeds were removed (expressed in days), which was used to compare the rates of seed removal in both types of vegetation.

### ﻿Statistical analysis

All statistical analyses were conducted in SPSS 22.0 for Windows (IBM, Inc., Armonk, NY, USA). Before the data analysis, the data was tested for normality and equality of variance using the Kolmogorov–Smirnov and Homogeneity-of-variance tests. Data were treated with respective nonparametric tests depending on they did not meet the assumptions of normality. A Cox regression was used to analyze the seed survival rates, factoring in in different types of habitats and seeds. The Kruskal–Wallis *H* test (nonparametric test) was used to compare the significant differences among the different seed species. The Mann–Whitney *U* test (nonparametric test) was used to test the differences between the different habitats and different seed species. The data are represented as the mean ± SD. The values are considered statistically significant at *P* < 0.05.

## ﻿Results

### ﻿Seed survival curves

**Figure 1.**
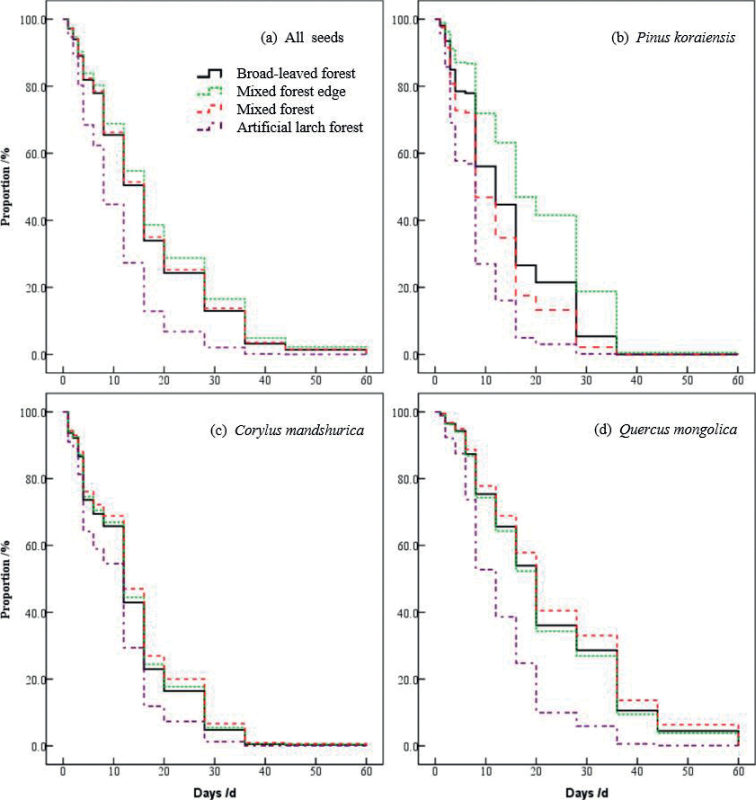
The survival curves of three kinds of seeds in different habitats in temperate forests of Northeast China.

The survival of seeds from different habitats was analysed to produce a survival curve. The survival curves of seeds showed the same trend, but the rates of consumption in different habitats varied (*W* = 111.958, df = 3, *P* < 0.001). The seeds were consumed the most quickly in the artificial larch forest. The rates of consumption in the other three habitats were very similar, and there were no significant differences in the degrees of differentiation among the seed survival curves (*W* = 3.526, df = 2, *P* = 0.172). In general, most of the early seeds were quickly devoured. After day 20, the consumption gradually decreased. Therefore, when the survival rate of the curve approached 20%, the curve became flat, and the trend of seed consumption decreased (Fig. 1).

The survival curves of three different types of seeds in the various habitats were analyzed. Each type of seed in all four habitats differed significantly (*P.koraiensis*: *W* = 88.400, df = 3, *P* < 0.001; *C.mandshurica*: *W* = 15.428, df = 3, *P* < 0.001; *Q.mongolica*: *W* = 54.848, df = 3, *P* < 0.001) (Fig. 1).

The most readily consumed type of seed was that of *P.koraiensis* in the artificial larch forest, followed by the mixed forest, then the broad-leaved forest, and finally, the mixed-forest edge. There were significant degrees of differentiation among the seed survival curves (*W* = 38.838, df = 2, *P* < 0.001), and the seed survival curves in different habitats differed significantly from each other (df = 1, *P* < 0.001) (Fig. 1).

The rates of consumption of *C.mandshurica* and *Q.mongolica* were also the highest in artificial larch forest. However, there were no significantly different survival curves in the broad-leaved forest, mixed-forest edge and mixed forest (*C.mandshurica*: *W* = 1.090, df = 2, *P* = 0.580; *Q.mongolica*: *W* = 2.109, df = 2, *P* = 0.348) (Fig. 1).

### ﻿Seed consumption time of the rodents

The seeds were discovered 1 d after they were released in the artificial larch forest and mixed forest. In contrast, the seeds were discovered 3 d after they were released in the secondary broad-leaved forest and mixed-forest edge. Rodents found the seeds in the artificial larch forest in a shorter average time than those in the other types of forests. The average earliest discovery time was 1.4 ± 0.9 d (1–3 d). The average earliest discovery time in all the other three habitats exceeded 7 d, 7.0 ± 3.0 d (3–12 d) in the broad-leaved forest, 7.5 ± 5.3 d (3–16 d) at the mixed-forest edge and 7.75 ± 9.4 d (1–28 d) in the mixed forest. Pairwise comparisons showed that the average time at which the food was first spotted differed significantly between the other habitats (*Z* = −2.363, *P* < 0.05). (Broad leaf forest: *Z* = −2.836, *P* < 0.01; mixed-forest edge: *Z* = −2.723, *P* < 0.05; mixed forest: *Z* = −1.853, *P* < 0.05). However, there was no difference in the average time for the discovery of first food in the other three habitats (*P* > 0.05).

### MRT of the seeds

When the seed species and habitat were not distinguished, the MRT was distributed around the seeds at 14.24 ± 10.53 d (1–60 d). In addition, there were significant differences in the MRT among different habitats (*χ*^2^ = 10.789, *P* < 0.05).

The MRT was shortest in the artificial larch forest at 7.67 ± 6.80 d (1–28 d). In contrast, the MRT in the broad-leaved forest was the longest at 17.52 ± 12.91 d (4–60 d). Pairwise comparisons showed that there were significant differences in the MRT between the artificial larch forest and the other habitats (broad-leaved forest: *Z* = −3.127, *P* < 0.01; mixed-forest edge: *Z* = −2.661, *P* < 0.01; mixed forest: *Z* = −2.459, *P* < 0.05). However, there were no differences in the MRT between the broad-leaved forest and mixed-forest edge (*Z* = −0.210, *P* > 0.05), the broad-leaved forest and mixed forest (*Z* = −0.499, *P* > 0.05), and the mixed-forest edge and mixed forest (*Z* = −0.097, *P* > 0.05) (Table 1).

**Table 1.** Median removal time of three kinds of seeds in different habitats in temperate forests of north-eastern China.

Habitats	Median removal time (Range; d)			
	All seeds	*P.koraiensis*	*C.mandshurica*	*Q.mongolica*
Broad-leaved forest	17.52 ± 12.91 (4–60)	13.71 ± 7.25 (8–28)	13.43 ± 7.89 (4–28)	25.43 ± 18.21 (6–60)
Mixed-forest edge	15.24 ± 9.35 (3–36)	17.17 ± 10.59 (3–36)	12.67 ± 5.89 (4–20)	16.00 ± 12.25 (6–36)
Mixed forest	14.78 ± 9.61 (1–36)	10.38 ± 9.23 (2–28)	12.63 ± 8.47 (1–28)	22.29 ± 7.61 (12–36)
Artificial larch forest	7.67 ± 6.80 (1–28)	6.00 ±2.74 (3–8)	6.20 ± 6.06 (1–16)	10.80 ± 9.96 (2–28)

Analyses of the survival curve of the three types of seeds in the different habitats showed that the fates of different seeds varied (Table 2). The rate of consumption of *P.koraiensis* seeds reached 96.70%; 99.09% of the *C.mandshurica* seeds were consumed, and 93.07% of the *Q.mongolica* seeds were consumed. More *Q.mongolica* seeds were intact *in situ* in various habitats, 11.43% in the broad-leaved forest, 8.00% in the mixed-forest edge, 4.29% in the mixed forest and 4.00% in the artificial larch forest.

**Table 2.** Statistics of three kinds of seeds with different fates in different habitats in temperate forests of Northeast China (unit: %).

Fate of seeds	Broad-leaved forest			Mixed-forest edge			Mixed forest			Artificial larch forest			All habitats		
	*P.koraiensis*	*C.mandshurica*	*Q.mongolica*	*P.koraiensis*	*C.mandshurica*	*Q.mongolica*	*P.koraiensis*	*C.mandshurica*	*Q.mongolica*	*P.koraiensis*	*C.mandshurica*	*Q.mongolica*	*P.koraiensis*	*C.mandshurica*	*Q.mongolica*
IS	0.71	0	11.43	12.50	0	8.00	0	0.63	4.29	0	3.00	4.00	3.30	0.91	6.93
PS	59.29	22.86	36.43	18.33	2.50	19.00	24.37	7.50	43.57	19.00	3.00	59.00	30.25	8.96	39.50
PR	5.00	10.71	15.00	10.00	13.33	25.00	17.50	14.38	19.29	41.00	19.00	27.00	18.38	14.35	21.57
IR	0	2.14	12.86	0.83	5.00	4.00	1.25	2.50	6.43	4.00	8.00	3.00	1.52	4.41	6.57
HR	14.29	37.86	5.71	3.34	21.67	20.00	25.63	32.50	5.00	11.00	19.00	1.00	13.56	27.76	7.93
MR	20.71	26.43	18.57	55.00	57.50	24.00	31.25	42.50	21.43	25.00	48.00	6.00	32.99	43.61	17.50
Consumption	99.29	87.50	100	100	96.70	100	100	99.38	97.00	90.09	88.57	92.00	95.72	96.00	93.07
Predation	64.29	33.57	51.43	28.33	15.83	44.00	41.87	21.88	62.86	60.00	22.00	86.00	48.63	23.31	61.07
Dispersal	35.00	66.43	37.14	59.17	84.17	48.00	58.13	77.50	32.86	40.00	75.00	10.00	48.07	78.75	32.00

In contrast to the other habitats, there was less predation of the three types of seeds at the mixed-forest edge, and the most seeds were dispersal. The rates of predation of the *P.koraiensis*, *C.mandshurica*, and *Q.mongolica* seeds were 28.33%, 15.83%, and 44.00%, respectively, and 59.17%, 84.17%, and 48.00% of the seeds were dispersed, respectively.

**Figure 2.**
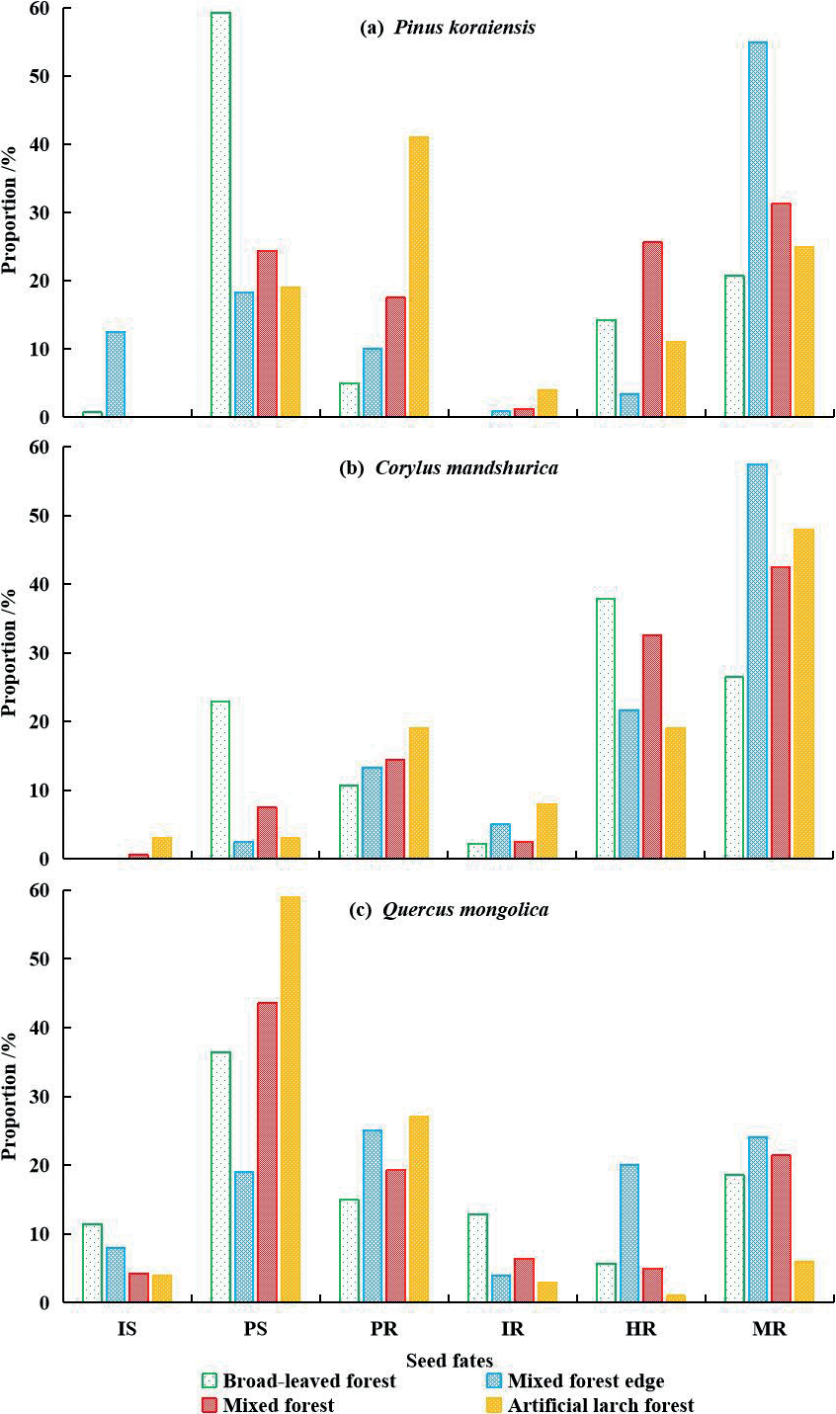
Statistics on the fate of three kinds of seeds in different habitats in temperate forests of Northeast China. Abbreviations: IS-Intact in situ, PS-Predation in situ, PR-Predation after removal, IR-Intact after removal, HR-Hoarded after removal, MR-Missing after removal.

In the broad-leaved forest and artificial larch forest, the rates of predation of the *P.koraiensis* seeds exceeded 60% and were higher than the dispersal rates, which were 35.00% and 40.00%, respectively. In contrast, the results were the opposite in the mixed-forest edge and mixed forest with the rate of dispersal of the *P.koraiensis* seeds exceeding 60%, which was much higher than the rates of predation of 28.33% and 41.83%, respectively (Fig. 2).

The dispersal rates of *C.mandshurica* were higher than the predation rates in all the habitats. The dispersal rate was the lowest in the broad-leaved forest (66.43%) and the largest at the mixed-forest edge (84.17%) (Fig. 2).

The predation rate of *Q.mongolica* was 44.00% in the mixed-forest edge, while it exceeded 50.0% in the other three types of habitats. It reached 86% in the artificial larch forest. However, the dispersal rate was only 10.00% in the artificial larch forest and approximately 35.00% in the other three types of habitats (Fig. 2).

**Figure 3.**
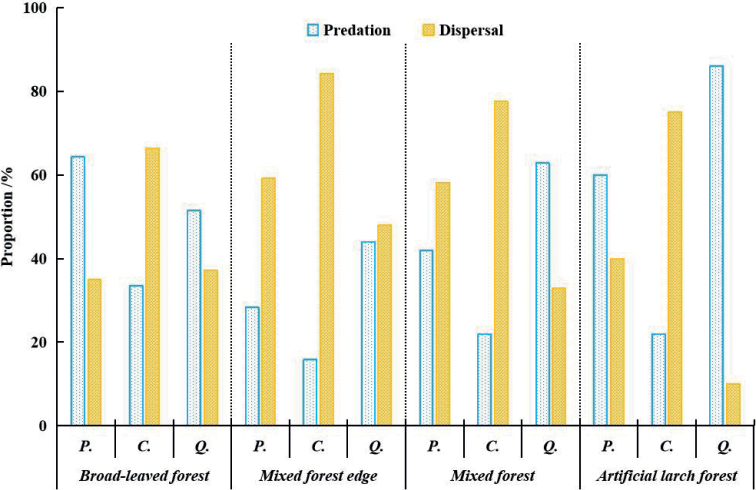
Predation rate and dispersal rate of three kinds of seeds in different habitats in temperate forests of Northeast China. Abbreviations: *P.*- *Pinuskoraiensis*, *C.*- *Corylusmandshurica*, *Q.*- *Quercusmongolica*.

When the habitats were not distinguished, the rate of predation of *P.koraiensis*, *C.mandshurica*, and *Q.mongolica* was 48.62%, 23.32%, and 61.07%, respectively. The dispersal rate was 48.07%, 75.78%, and 32.00%, respectively (Fig. 3).

### ﻿Dispersal of the seeds

The average dispersal distances of all the seeds were less than 6 m, and the longest distance recorded was 18.66 m. The dispersal distances and burial depths differed significantly among the four types of habitats (distance: *χ*^2^ = 24.149, *P* < 0.001; depth: *χ*^2^ = 24.334, *P* < 0.001) (Table 3). The distance of seed dispersal was primarily distributed within 1–6 m. Analyses of the statistical frequency indicated that the dispersal distances of seeds in the broad-leaved forest were consistent with those in the artificial larch forest. Approximately 44% of the seeds were dispersed within 1–3 m, while approximately 28% of the seeds were dispersed within 3–6 m. In the artificial larch forest, 16.22% were dispersed less than 1 m, and 10.58% were dispersed within 6–9 m in the broad-leaved forests. The rest of the distances were less than 10%.

**Table 3.** Dispersal distance and depth of burial of the seeds in different habitats in temperate forests of northeastern China.

Habitats	Dispersal distance (m)				Burial depth (cm)			
	All seeds	*P.koraiensis*	*C.mandshurica*	*Q.mongolica*	All seeds	*P.koraiensis*	*C.mandshurica*	*Q.mongolica*
Broad-leaved forest	4.10 ± 3.83	7.19 ± 6.06	3.38 ± 2.30	3.31 ± 3.38	1.53 ± 0.45	1.40 ± 0.31	1.61 ± 0.47	1.10 ± 0.42
Mixed-forest edge	5.31 ± 4.12	4.98 ± 1.48	4.89 ± 4.19	6.11 ± 4.81	1.18 ± 0.48	1.17 ± 0.29	0.94 ± 0.34	1.39 ± 0.52
Mixed forest	5.47 ± 3.55	6.09 ± 4.24	5.29 ± 2.90	1.80 ± 1.60	1.31 ± 0.36	1.23 ± 0.36	1.36 ± 0.35	–
Artificial larch forest	3.39 ± 3.12	3.40 ± 3.88	3.61 ± 2.98	2.13 ± 1.36	1.77 ± 0.68	1.00 ± 0.23	2.17 ± 0.48	1.50 ± 0.31

**Figure 4.**
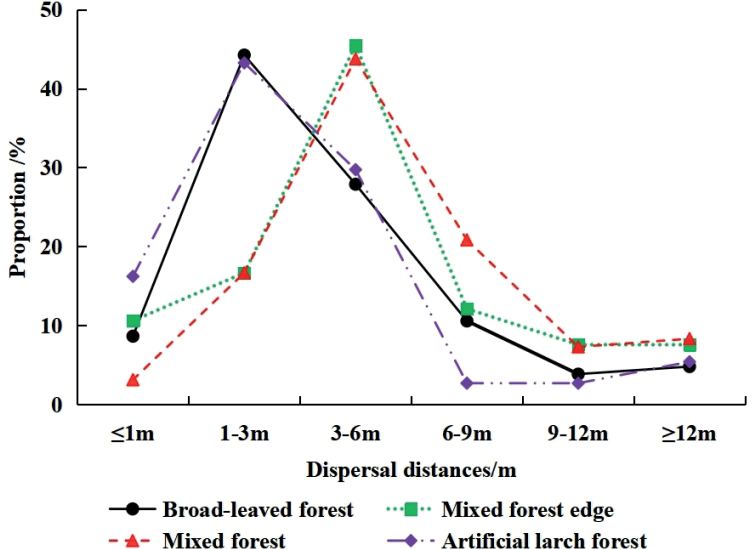
Dispersal distance of the seeds in different habitats.

The dispersal distances of seeds at the mixed-forest edge were consistent with those in the mixed forest. All showed the largest proportion of distances between 3 and 6 m, which accounted for approximately 44%. Dispersal distances of 1–3 m were close to accounting for 17%, while the distance of 6–9 m accounted for 20.83% in the mixed forest, and 12.12% at the mixed-forest edge. The proportion of distances greater than 9 m was less than 10% in both habitats (Fig. 4).

There were significant differences in the dispersal distance between the broad-leaved forest and mixed-forest edge (distance: *Z* = −2.566, *P* < 0.001; depth: *Z* = −3.589, *P* < 0.001), between the broad-leaved forest and mixed forest (distance: *Z* = −3.949, *P* < 0.001; distance: *Z* = −3.341, *P* < 0.001), and between the mixed forest and artificial larch forest (distance: *Z* = −3.811, *P* < 0.001; depth: *Z* = −3.077, *P* < 0.001). The differences in separate comparisons among the other habitats were found to lack significance according to the Mann–Whitney *U* test.

The was no significant difference in the dispersal distances of *P.koraiensis* in the four habitats. There were significant differences in the burial depth of *P.koraiensis* in the four habitats (distance: *χ*^2^ = 6.895, *P* = 0.075; depth: *χ*^2^ = 10.151, *P* < 0.05). Both the dispersal distances and burial depth of *C.mandshurica* differed significantly in the four habitats (distance: *χ*^2^ = 16.353, *P* < 0.001; depth: *χ*^2^ = 45.863, *P* < 0.001). The dispersal distances of *Q.mongolica* varied significantly in different habitats, but the difference in burial depth was not significant (distance: *χ*^2^ = 10.306, *P* < 0.05; depth: *χ*^2^ = 1.543, *P* = 0.462).

The burial depth of *C.mandshurica* and the dispersal distances of *Q.mongolica* differed significantly between the broad-leaved forest and mixed-forest edge (*C.mandshurica*: *Z* = −4.413, *P* < 0.001; *Q.mongolica*: *Z* = −2.430, *P* < 0.05).

A comparison of the broad-leaved forest and mixed forest indicated that the burial depth of *P.koraiensis* and the dispersal distance and burial depth of *C.mandshurica* differed significantly (depth of *P.koraiensis*: *Z* = −2.060, *P* < 0.05; distance of *C.mandshurica*: *Z* = −3.985, *P* < 0.001; depth of *C.mandshurica*: *Z* = −2.910, *P* < 0.05).

The broad-leaved forest and the artificial larch forest showed significant differences in the dispersal distance and burial depth of *P.koraiensis*. The burial depth of *C.mandshurica* showed significant differences (distance of *P.koraiensis*: *Z* = −2.314, *P* < 0.05; depth of *P.koraiensis*: *Z* = −2.892, *P* < 0.01; depth of *C.mandshurica*: *Z* = −2.910, *P* < 0.05).

There were significant differences in the dispersal distance of the *P.koraiensis* seeds between the mixed-forest edge and the artificial larch forest and the burial depth of *C.mandshurica* and dispersal distance of *Q.mongolica* (distance of *P.koraiensis*: *Z* = −2.056, *P* < 0.05; depth of *C.mandshurica*: *Z* = −4.833, *P* < 0.01; distance of *Q.mongolica*: *Z* = −2.204, *P* < 0.05).

Comparing the dispersal distance of *P.koraiensis* with that of *C.mandshurica* indicated that there were significant differences. In addition, there were differences in the burial depth of *C.mandshurica* between the mixed forest and the artificial larch forest (distance of *P.koraiensis*: *Z* = −2.401, *P* < 0.05; distance of *C.mandshurica*: *Z* = −2.770, *P* < 0.01; depth of *C.mandshurica*: *Z* = −5.046, *P* < 0.001).

## ﻿Discussion

### ﻿Factors that influence the rodent feeding strategies

The food availability of rodents in natural environments not only depends on their own feeding input but is also affected by various factors in the habitat (Vander Wall 2001; Lichti et al. 2015; Li et al. 2018, 2021). The availability of food resources (amount and form of distribution) is a central factor that influences the feeding strategy of rodents, which is an adaptation to the changes in food resources. The amount of food available to the rodents depends on the probability of encountering it, and the rodents adjust their feeding strategy by weighing the costs, such as time invested and search range, and the benefits in search for food based on the potential abundance and distribution of food resources in the environment (Vander Wall 2010; Wang et al. 2012). The niche breadth theory postulates that the width of ecological niche increases and generalizes that when there are few available resources, the animal decreases its search for the current resource and specializes where there are abundant available resources (Yang et al. 2011). Thus, foragers shift from selective to opportunistic feeding behaviour as the availability of food decreases.

### ﻿Habitat differences in rodent storage strategies

Different habitats have varying characteristics, and the differences in habitat characteristics affect the composition and structure of plant communities, spatial and temporal patterns, seclusion conditions, and food resources in the habitat (Xiao et al. 2005, 2006; Chang et al. 2008; Vander Wall 2010), which all affect the odds of animals encountering seeds. The amount of food resources and their distribution in the habitat leads to differences in the time and energy required to search for and process food, and animals will change their range of activity depending on the availability of food resources. Habitat heterogeneity also alters intra- or interspecific competition patterns by affecting the density and distribution of rodents, which, in turn, affects the ability of rodent to feed on and disperse seeds (Cao et al. 2016; Zhang et al. 2017).

The results showed that there was a significant difference in when the food was first discovered and the rate and time of consumption of rodents in the four habitats, which reflects the influence of different habitat characteristics. The ratio of feeding to dispersal is the result of the trade-off between the food availability, competition, or predation risk of rodents and the result of optimizing resource acquisition. The results of this study suggest that the habitat characteristics of the mixed-forest edge and larch plantation seem to be relatively special. They appear to be quite different from most forest habitats in vegetation composition and structure, community appearance, and understory microhabitat characteristics, so there are many obvious differences in the study results.

The seed residuals were higher; fewer seeds were taken; the rate of dispersal was the highest, and the consumption time was the slowest in mixed-forest edge habitats. This could be owing to the effects of high species diversity and interspecific competition in the community edge effect, and the low number of trees that result in poorly concealed open habitats and a high risk of predation. Animals adopt strategies to carry stored seeds to reduce the competition and risk of predation, and at a higher risk of predation, they adjust their behavioural strategies, such as becoming increasingly vigilant and reducing foraging (Jonsson et al. 2000; Jansen et al. 2006; Randall and Boltas 2011). It is definitely a sound strategy to sacrifice some food resources to ensure safety.

In the artificial larch forest, not only do animals meet seeds at the earliest, but 50% of the consumption time is significantly shorter. Thus, it takes only approximately half the time of other habitats to find food. Since the vegetation species is single and dense with high cover, the branches are interspersed and good at concealing the rodents. However, the food resources are not abundant, and rapid feeding or dispersal is an effective way to occupy more resources. Such a feeding response is consistent with the rapid isolation hypothesis.

The slowest rate of *Q.mongolica* depletion in the broad-leaved forest could be mostly owing to the fact that *Q.mongolica* is the dominant species in the habitat vegetation, and the seed resources are abundant on the scattered surface. Thus, the animals prefer these seeds when there are multiple seeds that are equally available.

## ﻿Conclusions

Rodent feeding and storage strategies differ significantly between habitats. Different habitats are significantly heterogeneous, which leads to significantly different strategies for seed consumption in rodents. The quickest consumption occurs in the artificial larch forest, the slowest at the mixed-forest edge, and the rate of consumption in the broad-leaved forest and mixed forest are close to that of the mixed-forest edge.

Rodents can identify different seed properties of the sympatric distribution and form distinct feeding preferences. Rodents adopt different feeding or storage methods, causing the seeds to have different fates. The seeds of *Q.mongolica* are the mostly strongly preferred for consumption. More seeds of *P.koraiensis* are eaten and stored, and the seeds of *C.mandshurica* are stored the most frequently.
